# Papel da Rigidez Aórtica na Previsão da Resposta aos Inibidores da Fosfodiesterase-5 no Tratamento da Disfunção Erétil

**DOI:** 10.36660/abc.20230514

**Published:** 2024-03-12

**Authors:** Ömer Faruk Çiçek, Halil Ferat Öncel, Remzi Salar

**Affiliations:** 1 Mehmet Akif Inan Education and Research Hospital Department of Cardiology Sanliurfa Turquia Department of Cardiology, Mehmet Akif Inan Education and Research Hospital, Sanliurfa – Turquia; 2 Mehmet Akif Inan Education and Research Hospital Department of Urology Sanliurfa Turquia Department of Urology, Mehmet Akif Inan Education and Research Hospital, Sanliurfa – Turquia

**Keywords:** Disfunção Erétil, Inibidores da Fosfodiesterase 5, Ecocardiografia

## Abstract

**Fundamento:**

Sabe-se que a rigidez aórtica (RA) aumenta em pacientes com disfunção erétil (DE). Os inibidores da enzima fosfodiesterase tipo 5 (PDE-5) são usados no tratamento da DE, e as respostas dos pacientes a esse tratamento podem variar.

**Objetivos:**

Nosso objetivo foi investigar o papel da RA na previsão da resposta de pacientes planejados para tomar inibidores da enzima PDE-5 devido à DE.

**Métodos:**

Um total de 96 pacientes do sexo masculino com DE foram incluídos no estudo. O questionário do Índice Internacional de Função Erétil (IIEF) foi utilizado para avaliar a presença e gravidade da DE e a resposta ao tratamento. A ecocardiografia transtorácica foi utilizada para avaliar RA.

**Resultados:**

Houve diferença estatisticamente significativa entre os valores de deformação aórtica e distensibilidade aórtica dos grupos de estudo (p<0,001). O escore delta IIEF apresentou alto nível de correlação positiva com a deformação aórtica (p<0,01, r=0,758) e um nível moderado de correlação positiva com a distensibilidade aórtica (p<0,01, r=0,574).

**Conclusão:**

Determinamos que em pacientes com DE, a deformação aórtica e a distensibilidade aórtica medidas de forma não invasiva por meio de ecocardiografia transtorácica são parâmetros importantes na previsão da resposta dos pacientes à terapia com inibidores da PDE-5.

**Figure:**
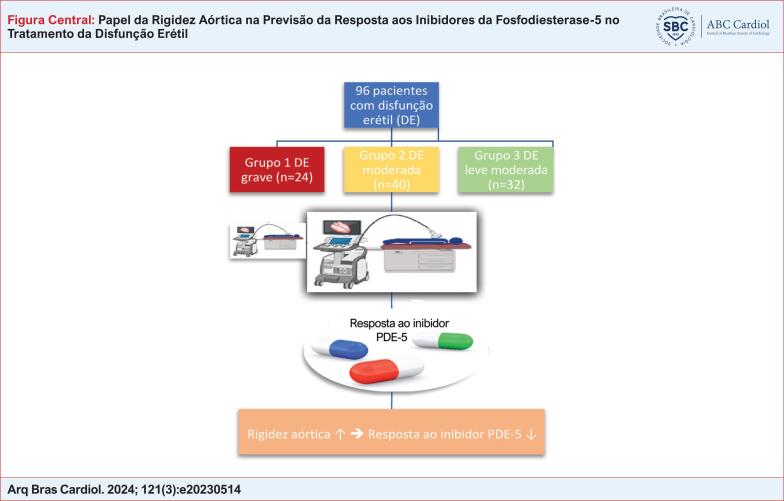


## Introdução

A disfunção endotelial ocorre devido a uma diminuição dependente do endotélio no óxido nítrico (NO), resultando na progressão da aterosclerose.^1 , 2^ Os efeitos da aterosclerose podem ser avaliados através da rigidez aórtica (RA). Entre as condições clínicas em que a RA está aumentada estão a doença arterial coronariana, diabetes mellitus, hipertensão e distúrbios da tireoide. O aumento da RA leva à hipertensão sistólica, hipertrofia ventricular esquerda e deterioração da perfusão coronariana, aumentando assim o risco de doença cardiovascular (DCV).^3^ Além de métodos simples como pressão de pulso, são usados para avaliar RA muitos métodos pouco práticos e que exigem equipamentos caros.^4^ A ecocardiografia transtorácica é um método não invasivo que também pode avaliar a variabilidade pulsátil, tensão e distensibilidade na aorta.^5^

A interrupção da via do NO causa disfunção endotelial, o que pode ser importante para desencadear eventos vasculares, uma vez que a bioatividade prejudicada do NO é preditiva da atividade da doença aterosclerótica. O aumento da RA também é um indicador do desenvolvimento de doença arterial coronariana, doença cerebrovascular e doença arterial periférica.^6 - 8^ Além disso, a disfunção que ocorre no endotélio vascular periférico também é um indicador de disfunção erétil (DE). A DE foi definida como a incapacidade persistente de atingir e manter uma ereção suficiente para permitir um desempenho sexual satisfatório.^9^ O NO é um dos mediadores mais importantes na fisiologia da ereção e é conhecido por desempenhar um papel importante na RA.^10 - 12^ Além disso, estudos anteriores relataram que a RA está aumentada em pacientes com DE em comparação com aqueles sem DE.^1 , 13 - 16^ Os inibidores da enzima fosfodiesterase tipo 5 (PDE-5) usados no tratamento da DE proporcionam ereção peniana aumentando a concentração de NO no tecido muscular liso e facilitam o esvaziamento da bexiga, causando relaxamento no colo da bexiga.^17^ As fosfodiesterases (PDEs) são uma superfamília de enzimas que catalisam a hidrólise de monofosfatos de nucleotídeos, o monofosfato de adenosina cíclico (AMPc) e o monofosfato de guanosina cíclico (cGMP) aos seus monofosfatos 5' correspondentes.^18^ Até o momento, vários PDEs, incluindo PDE-5, PDE-7, PDE-8, PDE-9, PDE-10 e PDE-11, foram identificados e caracterizados.^19 , 20^ A enzima PDE-5 é amplamente distribuída por todo o corpo, incluindo o coração e os vasos sanguíneos. Os inibidores da PDE-5 são inibidores seletivos da enzima PDE-5 que catalisam a hidrólise do cGMP, um potente vasodilatador e doador de NO, em seus metabólitos correspondentes (monofosfatos).^21^

À luz das informações fornecidas acima, nosso objetivo foi investigar o papel da RA na previsão da resposta ao tratamento em pacientes programados para iniciar terapia com inibidor da PDE-5 devido à DE.

## Material e método

Foi recebida para o protocolo do estudo a aprovação do Conselho de Revisão Institucional. O estudo foi desenhado prospectivamente e incluiu 96 pacientes do sexo masculino com idade superior a 18 anos que se apresentaram no ambulatório de urologia do Hospital de Treinamento e Pesquisa Sanliurfa Mehmet Akif Inan e foram planejados para iniciar o tratamento com inibidor PDE-5 de acordo com sua anamnese, achados do exame físico e níveis de glicemia de jejum (FBG), colesterol total, triglicerídeos, colesterol de lipoproteína de baixa densidade (LDL), testosterona total, hormônio estimulador da tireoide (TSH) e prolactina. Os pacientes não tinham doença cardíaca conhecida, diabetes ou hipertensão. Amostras de sangue venoso de todos os pacientes participantes do estudo foram coletadas em tubos de bioquímica de gel e analisadas. A presença e gravidade da DE e a resposta ao tratamento foram avaliadas usando o Índice Internacional de Função Erétil (IIEF) de 15 itens, um questionário adotado globalmente desenvolvido por Rosen et al. De acordo com os escores do IIEF, os pacientes foram classificados nos seguintes grupos: Grupo 1 (0-10, grave); Grupo 2 (11-16 anos, DE moderada); Grupo 3 (17-21, DE leve a moderada); Grupo 4 (22-25, DE leve); e Grupo 5 (26-30, sem DE). A resposta dos pacientes ao tratamento foi avaliada pelo IIEF um mês após o início dos inibidores da PDE-5.

A avaliação ecocardiográfica dos grupos de pacientes foi realizada por um cardiologista que desconhecia os achados clínicos e laboratoriais. O exame ecocardiográfico foi realizado no Vingmed System 5 (Vivid S5, GE, Horten, Noruega) com transdutor de 2,5-3,5 MHz. A análise quantitativa com ecocardiografia modo M foi feita nas imagens paraesternais do eixo longo de acordo com os dados da Sociedade Americana de Ecocardiografia.^22^

As funções sistólica e diastólica do ventrículo esquerdo foram avaliadas por meio de ecocardiografia bidimensional (2D) padrão, ecocardiografia modo M, ecocardiografia de onda pulsada (OP) e ecocardiografia Doppler tecidual. O diâmetro atrial esquerdo, a espessura do septo interventricular (SIV), a espessura da parede posterior, o diâmetro diastólico final do ventrículo esquerdo (DDVE) e o diâmetro sistólico final do ventrículo esquerdo (DSVE) foram medidos, e a fração de ejeção (FE) foi calculada usando ecocardiografia 2D e ecocardiografia modo M. Os registros da aorta ascendente foram feitos no modo M sob a orientação da ecocardiografia 2D. Os registros da aorta ascendente no modo M foram feitos 3 cm acima da valva aórtica. Os diâmetros aórticos foram calculados tomando as distâncias entre as bordas internas das paredes anterior e posterior da aorta na sístole e na diástole. O diâmetro sistólico da aorta foi medido quando a válvula aórtica estava na posição totalmente aberta. O diâmetro diastólico da aorta foi medido simultaneamente com o pico do QRS nos registros do eletrocardiograma. As medições foram realizadas em cinco batimentos consecutivos e sua média foi obtida.

Os valores de pressão arterial sistólica (PAS) e pressão arterial diastólica (PAD) foram medidos com um esfigmomanômetro externo. A pressão de pulso foi calculada como PAS menos PAD. A deformação aórtica (%) foi determinada como (diâmetro sistólico aórtico – diâmetro diastólico) x100/diâmetro diastólico aórtico. Por último, a distensibilidade aórtica (106 cm^2^/dyn) foi calculada como (2 x deformação aórtica)/pressão de pulso.

### Análise estatística

A análise dos dados foi realizada com o programa Statistical Package for the Social Sciences (SPSS) v. A conformidade das variáveis de medida contínua com a distribuição normal foi determinada por meio de valores de assimetria e curtose, além do teste de Shapiro-Wilk. As estatísticas descritivas foram expressas como média ± desvio padrão para variáveis contínuas e números e porcentagens de observações para variáveis categóricas. A homogeneidade das características obtidas pelas medidas entre os grupos controle e pacientes foi investigada pelo teste de Levene, e a significância das diferenças foi determinada pelo teste de análise de variância unidirecional. A análise de correlação de Pearson foi utilizada para calcular os coeficientes de correlação. Um valor de p <0,05 foi considerado estatisticamente significativo.

## Resultados

Nenhuma diferença significativa foi observada entre os grupos de DE em termos de características basais ( Tabela 1 ).

**Tabela 1:** Características basais dos grupos de DE

	Grupo 1 (DE grave) n = 24	Grupo 2 (DE moderada) n = 40	Grupo 3 (DE leve a moderada) n = 32	Valor de p
Idade	49,83±8,69	48,2±10,39	45,18±752	0,15
Glicose sanguínea em jejum (mg/dl)	93,25±8,84	98,37±15,79	96,96±8,93	0,27
Creatinina (mg/dl)	0,90±0,11	0,95±0,18	0,89±0,14	0,18
Colesterol total (mg/dl)	197,16±18,65	189,62 ±27,22	185,90±20,76	0,11
Triglicerídeos (mg/dl)	153±43,63	154±70,94	145±38,76	0,76
Colesterol LDL (mg/dl)	134,87±19,65	126,25 ±24,23	125,03±17	0,17
Testosterona total (ng/ml)	4,94±1,52	4,79±1,34	4,81±1,22	0,90
Hormônio estimulador da tireoide	2,30±0,55	2,69±0,67	2,05±0,46	0,14
Hormônio prolactina	13,43±4,39	12,02±5,50	11,63±3,23	0,32
Pressão arterial sistólica (mmHg)	111,66±12,03	108,30 ± 9,11	109,70±9,91	0,41
Pressão arterial diastólica (mmHg)	82,83±9,14	79±4,96	80,62±8,95	0,15
Fumante (%)	20,83%	17,5%	15,62%	0,68

DE: disfunção erétil; LDL: lipoproteína de baixa densidade.

Entre os parâmetros calculados pela ecocardiografia modo M, o diâmetro do átrio esquerdo, a espessura do SIV, o DDVE, o DSVE, a espessura da parede posterior e a FE não diferiram estatisticamente significativamente entre os grupos ( Tabela 2 ).

**Tabela 2:** Resultados ecocardiográficos convencionais dos grupos de DE

	Grupo 1 (DE grave) n = 24	Grupo 2 (DE moderada) n = 40	Grupo 3 (DE leve a moderada) n = 32	Valor de p
AE (mm)	31,37±1,17	31,67±1,09	32,0±1,43	0,15
SIV (mm)	10,37±0,82	10,12±0,72	10,25±0,67	0,41
DDVE (mm)	44,37±1,83	43,8±1,87	44,12±2,16	0,51
DSVE (mm)	28,1±1,09	28,6±1,12	28,2±1,56	0,23
PP (mm)	9,8±1,09	9,9±0,70	9,1±1,37	0,08
FE (%)	63,5±1,53	62,7±2,31	63,3±1,01	0,14

DE: disfunção erétil; DDVE: diâmetro diastólico final do ventrículo esquerdo; DSVE: diâmetro sistólico final do ventrículo esquerdo; AE: átrio esquerdo; SIV: septo interventricular; PP: parede posterior; FE: fração de ejeção.

Os resultados dos parâmetros elásticos aórticos ecocardiográficos são apresentados na Tabela 3 . Os valores de strain aórtico do Grupo 1, Grupo 2 e Grupo 3 foram 8,38, 11,63 e 14,57, respectivamente, indicando diferenças estatisticamente significativas (p < 0,001). A distensibilidade aórtica apresentou variações estatisticamente significativas entre os diferentes grupos de disfunção erétil (DE), com valores de 0,60, 0,90 e 1,05 registrados para o Grupo 1, Grupo 2 e Grupo 3, respectivamente. e 1,05 no Grupo 3 (p < 0,001). Da mesma forma, houve diferenças estatisticamente significativas nos valores do diâmetro sistólico e diastólico da aorta entre os grupos de DE (p < 0,001).

**Tabela 3:** Parâmetros ecocardiográficos elásticos aórticos dos grupos DE

	Grupo 1 (DE grave) n = 24	Grupo 2 (DE moderada) n = 40	Grupo 3 (DE leve a moderada) n = 32	Valor de p
DSAo (mm)	28±0,83	30,1 ±1,39	31,5±0,71	<0,001
DDAo (mm)	25,83±0,70	26,9±1,31	27,5±0,71	<0,001
Distensão aórtica (%)	8,38±1,45	11,93±2,45	14,57±2,01	<0,001
Distensibilidade aórtica 10-3 cm^2^/dyn	0,60±0,18	0,90±0,38	1,05±0,28	<0,001

DE: disfunção erétil; DSAo: diâmetro sistólico aórtico; DDAo: diâmetro diastólico aórtico.

Quando foram examinadas as alterações pós-tratamento nos grupos de pacientes de acordo com a gravidade da DE, havia 24 pacientes no Grupo 1, 40 pacientes no Grupo 2 e 32 pacientes no Grupo 3 antes do tratamento, enquanto havia quatro pacientes no Grupo 1, 24 pacientes no Grupo 2, 16 pacientes no Grupo 3, 32 pacientes no Grupo 4 e 20 pacientes no Grupo 5 após o tratamento ( Tabela 4 ).

**Tabela 4:** Distribuição dos pacientes entre os grupos de disfunção erétil antes e depois do tratamento

		Grupos pós-tratamento					Total
		Grupo 1	Grupo 2	Grupo 3	Grupo 4	Grupo 5	
**Grupos de pré-tratamento**	**Grupo 1**	4	16	4	0	0	24
	**Grupo 2**	0	8	12	20	0	40
	**Grupo 3**	0	0	0	12	20	32
**Total**		4	24	16	32	20	96

Grupo 1 (0-10, grave); Grupo 2 (11-16 anos, DE moderada); Grupo 3 (17-21, DE leve a moderada); Grupo 4 (22-25, DE leve); e Grupo 5 (26-30, sem DE).

A pontuação média do delta IIEF (diferença entre as pontuações IIEF pré-tratamento e pós-tratamento) foi determinada como 5,13. Embora tenha havido uma alta correlação positiva entre o escore delta IIEF dos pacientes e a deformação aórtica (p < 0,01, r = 0,758), uma correlação positiva moderada foi observada com a distensibilidade aórtica (p < 0,01, r = 0,574).

## Discussão

Neste estudo, nosso objetivo foi investigar o papel da RA na previsão da resposta ao tratamento em pacientes que iniciaram inibidores da PDE-5 para DE. A disfunção endotelial e a aterosclerose são os fatores etiológicos mais importantes nas DCV.^23^ Sabe-se que a aterosclerose afeta os vasos cavernosos mais cedo do que as artérias coronárias devido aos seus diâmetros menores. A disfunção endotelial também pode causar o desenvolvimento de DE; portanto, a DE é considerada uma manifestação precoce de doenças vasculares sistêmicas.^15 , 24 , 25^ Da mesma forma, a RA é utilizada como método não invasivo para detecção precoce de aterosclerose subclínica. A diminuição da elasticidade da aorta e o aumento da sua rigidez são aceitos como indicadores de alteração aterosclerótica.^26^

Neste estudo, descobrimos que os valores dos parâmetros de elasticidade aórtica, deformação aórtica e distensibilidade aórtica diminuíram paralelamente à gravidade da DE, enquanto a RA aumentou. Em estudo de Demirelli et al. examinando a relação entre a gravidade da DE e RA, houve um aumento na gravidade da DE à medida que a RA aumentou.^1^ É bem conhecido que o NO desempenha um papel importante na regulação da elasticidade arterial. Assim, a inibição da síntese de NO aumenta a RA. O NO é um dos mediadores mais importantes na fisiologia da ereção.^10 - 12^ O envolvimento do NO na etiologia da DE e da aterosclerose sugere uma relação entre essas duas condições. Estudos também demonstraram que pacientes com DE apresentam RA significativamente maior do que aqueles sem DE.^1 , 13 - 16^

Muitos estudos epidemiológicos demonstraram uma associação entre DE e DCV. Uma meta-análise revelou que homens com DE apresentavam um risco significativamente aumentado de DCV, doença coronariana e acidente vascular cerebral em comparação aos grupos de referência.^27 , 28^ Essas associações podem ser atribuídas à disfunção endotelial e à hipótese do tamanho da artéria, que propõe que a aterosclerose afeta todos os principais leitos vasculares na mesma extensão e que as artérias penianas com diâmetro menor que os vasos coronários e carótidos são afetadas mais cedo por placas ateroscleróticas do mesmo tamanho. A disfunção endotelial sistêmica pode fazer com que o pênis não seja capaz de regular o fluxo sanguíneo nos corpos cavernosos.^29^ O achado mais importante do nosso estudo foi a correlação entre os escores delta IIEF dos pacientes e a distensão e distensibilidade aórtica, com um alto nível de correlação positiva para o primeiro e uma correlação positiva moderada para o segundo. Isto sugere que a RA é um parâmetro que pode ser usado para prever a resposta ao tratamento antes do seu início em pacientes com DE.

Os inibidores da PDE-5 ocupam um lugar importante no tratamento da DE. Após o tratamento da DE, a resposta é avaliada pelo escore IIEF. O alto nível de correlação positiva entre o escore delta IIEF (ou seja, a diferença entre os escores IIEF pré-tratamento e pós-tratamento) e a deformação aórtica mostra que a RA pode ser avaliada com ecocardiografia transtorácica, que é facilmente aplicável na previsão da resposta dos pacientes com DE para inibidores da PDE-5 antes do início do tratamento. Além disso, como mencionado acima, uma vez que a DE pode ser um sinal precoce de doenças vasculares sistêmicas, uma avaliação cardíaca nesses pacientes pode auxiliar o médico na detecção de possíveis DCV e na previsão da resposta aos inibidores da enzima PDE-5.

As limitações do nosso estudo são que ele foi unicêntrico e o número de pacientes foi pequeno. Há necessidade de estudos multicêntricos com séries de casos maiores sobre esse assunto.

## Conclusão

Determinamos que em pacientes com DE, a deformação aórtica e a distensibilidade aórtica medidas de forma não invasiva por meio de ecocardiografia transtorácica são parâmetros importantes na previsão das respostas dos pacientes à terapia com inibidores da PDE-5.
