# Supplementary material for: Papel da Rigidez Aórtica na Previsão da Resposta aos Inibidores da Fosfodiesterase-5 no Tratamento da Disfunção Erétil
Source: Arq Bras Cardiol. 2024 Mar 12;121(3):e20230514. [Article in Portuguese] doi: 10.36660/abc.20230514 (PMC12092020; doi:10.36660/abc.20230514)
Supplement: Translation

# Translation: Role of Aortic Stiffness in Predicting Response to Phosphodiesterase-5 Inhibitors in the Treatment of Erectile Dysfunction

**Authors:** Ömer Faruk Çiçek, Halil Ferat Öncel, Remzi Salar

**Affiliations:** 1 Sanliurfa Turkey Department of Cardiology, Mehmet Akif Inan Education and Research Hospital, Sanliurfa – Turkey; 2 Sanliurfa Turkey Department of Urology, Mehmet Akif Inan Education and Research Hospital, Sanliurfa – Turkey

**Keywords:** Erectile Dysfunction, Phosphodiesterase 5 Inhibitors, Echocardiography

## Abstract

**Background:**

It is known that aortic stiffness (AS) increases in patients with erectile dysfunction (ED). Phosphodiesterase type-5 (PDE-5) enzyme inhibitors are used in the treatment of ED, and patients' responses to this treatment may vary.

**Objectives:**

We aimed to investigate the role of AS in predicting the response of patients planned to take PDE-5 enzyme inhibitors due to ED.

**Methods:**

A total of 96 male patients with ED were included in the study. The International Index of Erectile Function (IIEF) questionnaire was used to evaluate the presence and severity of ED and the response to treatment. Transthoracic echocardiography was used to evaluate AS.

**Results:**

There was a statistically significant difference between the aortic strain and aortic distensibility values of the study groups (p<0.001). The delta IIEF score had a high level of positive correlation with aortic strain (p<0.01, r=0.758) and a moderate level of positive correlation with aortic distensibility (p<0.01, r=0.574).

**Conclusion:**

We determined that in patients with ED, aortic strain and aortic distensibility measured non-invasively using transthoracic echocardiography are important parameters in predicting patients' response to PDE-5 inhibitor therapy.

## Introduction

Endothelial dysfunction occurs due to an endothelium-dependent decrease in nitric oxide (NO), resulting in the progression of atherosclerosis.^1 , 2^The effects of atherosclerosis can be evaluated using aortic stiffness (AS). Among the clinical conditions in which AS is increased are coronary artery disease, diabetes mellitus, hypertension, and thyroid disorders. Increased AS leads to systolic hypertension, left ventricular hypertrophy, and deterioration in coronary perfusion, thereby raising the risk of cardiovascular disease (CVD).^3^Apart from simple methods like pulse pressure, many methods, which are unpractical and warrant expensive equipment, are used to evaluate AS.^4^Transthoracic echocardiography is a non-invasive method that can also evaluate pulsatile variability, strain, and distensibility in the aorta.^5^

Disruption of the NO pathway causes endothelial dysfunction, which may be important for triggering vascular events as impaired NO bioactivity is predictive of atherosclerotic disease activity. Increased AS is also an indicator of the development of coronary artery disease, cerebrovascular disease, and peripheral artery disease.^6 - 8^In addition, dysfunction that occurs in the peripheral vascular endothelium is also an indicator of erectile dysfunction (ED). ED has been defined as the persistent inability to achieve and maintain an erection sufficient to allow satisfactory sexual performance.^9^NO is one of the most important mediators in the physiology of erection and is known to play an important role in AS.^10 - 12^Furthermore, previous studies have reported that AS is increased in patients with ED compared to those without ED.^1 , 13 - 16^Phosphodiesterase type-5 (PDE-5) enzyme inhibitors used in the treatment of ED both provide penile erection by increasing the NO concentration in smooth muscle tissue and facilitate the emptying of the bladder by causing relaxation in the bladder neck.^17^Phosphodiesterases (PDEs) are a superfamily of enzymes that catalyze the hydrolysis of nucleotide monophosphates, the cyclic adenosine monophosphate (cAMP), and the cyclic guanosine monophosphate (cGMP) to their corresponding 5' monophosphates.^18^To date, several PDEs, including PDE-5, PDE-7, PDE-8, PDE-9, PDE-10, and PDE-11, have been identified and characterized.^19 , 20^The PDE-5 enzyme is widely distributed throughout the body, including the heart and blood vessels. PDE-5 inhibitors are selective PDE-5 enzyme inhibitors that catalyze the hydrolysis of cGMP, a potent vasodilator and NO donor, to its corresponding metabolites (monophosphates).^21^

In light of the information given above, we aimed to investigate the role of AS in predicting the response to treatment in patients scheduled to start PDE-5 inhibitor therapy due to ED.

## Material and Method

Approval of the Institutional Review Board was received for the study protocol. The study was designed prospectively and included 96 male patients aged over 18 years who presented to the urology outpatient clinic of the Sanliurfa Mehmet Akif Inan Training and Research Hospital and were planned to start PDE-5 inhibitor treatment in line with their anamnesis, physical examination findings, and fasting blood glucose (FBG), total cholesterol, triglyceride, low-density lipoprotein (LDL) cholesterol, total testosterone, thyroid-stimulating hormone (TSH), and prolactin levels. The patients had no known cardiac disease, diabetes, or hypertension. Venous blood samples from all patients participating in the study were taken into gel biochemistry tubes and analyzed. The presence and severity of ED and the response to treatment were evaluated using the 15-item International Index of Erectile Function (IIEF), a globally adopted questionnaire developed by Rosen et al. According to the IIEF scores, the patients were classified into the following groups: Group 1 (0-10, severe); Group 2 (11-16, moderate ED); Group 3 (17-21, mild-moderate ED); Group 4 (22-25, mild ED); and Group 5 (26-30, no ED). The response of the patients to treatment was evaluated using the IIEF one month after PDE-5 inhibitors were started.

The echocardiographic evaluation of the patient groups was performed by a cardiologist who was blinded to the clinical and laboratory findings. The echocardiographic examination was performed using the Vingmed System 5 (Vivid S5, GE, Horten, Norway) with a 2.5-3.5 MHz transducer. Quantitative analysis with M-Mode echocardiography was made on parasternal long-axis images according to the data of the American Society of Echocardiography.^22^

Left ventricular systolic and diastolic functions were evaluated using standard two-dimensional (2D) echocardiography, M-mode echocardiography, pulsed-wave (PW) echocardiography, and tissue Doppler echocardiography. Left atrial diameter, interventricular septum (IVS) thickness, posterior wall thickness, left ventricular end-diastolic diameter (LVEDD), and left ventricular end-systolic diameter (LVESD) were measured, and the ejection fraction (EF) was calculated using 2D echocardiography and M-mode echocardiography. Ascending aortic recordings were taken using M-mode under the guidance of 2D echocardiography. M-mode ascending aorta recordings were made 3 cm above the aortic valve. Aortic diameters were calculated by taking the distances between the anterior and posterior wall inner edges of the aorta in systole and diastole. The systolic diameter of the aorta was measured when the aortic valve was in the fully open position. The diastolic diameter of the aorta was measured simultaneously with the peak of the QRS in the electrocardiogram recordings. Measurements were performed on five consecutive beats, and their average was taken.

Systolic blood pressure (SBP) and diastolic blood pressure (DBP) values were measured with an external sphygmomanometer. Pulse pressure was calculated as SBP minus DBP. Aortic strain (%) was determined as (aortic systolic diameter – diastolic diameter) x100/aortic diastolic diameter. Lastly, aortic distensibility (106 cm2/dyn) was calculated as (2 x aortic strain)/pulse pressure.

### Statistical analysis

Data analysis was performed with the Statistical Package for the Social Sciences (SPSS) v. 24 package program. The conformity of the continuous-measure variables to the normal distribution was determined using skewness and kurtosis values, as well as the Shapiro-Wilk test. Descriptive statistics were expressed as mean ± standard deviation for continuous variables and numbers and percentages of observations for categorical variables. The homogeneity of the characteristics obtained by measurements between the control and patient groups was investigated with the Levene test, and the significance of differences was determined using the one-way analysis of variance test. Pearson correlation analysis was used to calculate correlation coefficients. A p-value of <0.05 was considered statistically significant.

## Results

No significant difference was observed between the ED groups in terms of baseline characteristics ( Table 1 ).

**Table 1:** Baseline characteristics of the ED groups

	Group 1 (severe ED) n = 24	Group 2 (moderate ED) n = 40	Group 3 (mild-moderate ED) n = 32	p value
Age	49.83±8.69	48.2±10.39	45.18±752	0.15
Fasting blood glucose (mg/dl)	93.25±8.84	98.37±15.79	96.96±8.93	0.27
Creatinine (mg/dl)	0.90±0.11	0.95±0.18	0.89±0.14	0.18
Total cholesterol (mg/dl)	197.16±18.65	189.62 ±27.22	185.90±20.76	0.11
Triglyceride (mg/dl)	153±43.63	154±70.94	145±38.76	0.76
LDL cholesterol (mg/dl)	134.87±19.65	126.25 ±24.23	125.03±17	0.17
Total testosterone (ng/ml)	4.94±1.52	4.79±1.34	4.81±1.22	0.90
Thyroid-stimulating hormone	2.30±0.55	2.69±0.67	2.05±0.46	0.14
Prolactin hormone	13.43±4.39	12.02±5.50	11.63±3.23	0.32
Systolic blood pressure (mmHg)	111.66±12.03	108.30 ± 9.11	109.70±9.91	0.41
Diastolic blood pressure (mmHg)	82.83±9.14	79±4.96	80.62±8.95	0.15
Smoker (%)	20.83%	17.5%	15.62%	0.68

ED: erectile dysfunction; LDL: low-density lipoprotein.

Among the parameters calculated using M-mode echocardiography, left atrial diameter, IVS thickness, LVEDD, LVESD, posterior wall thickness, and EF did not statistically significantly differ between the groups ( Table 2 ).

**Table 2:** Conventional echocardiographic results of the ED groups

	Group 1 (severe ED) n = 24	Group 2 (moderate ED) n = 40	Group 3 (mild-moderate ED) n = 32	p value
LA (mm)	31.37±1.17	31.67±1.09	32.0±1.43	0.15
IVS (mm)	10.37±0.82	10.12±0.72	10.25±0.67	0.41
LVEDD (mm)	44.37±1.83	43.8±1.87	44.12±2.16	0.51
LVESD (mm)	28.1±1.09	28.6±1.12	28.2±1.56	0.23
PW (mm)	9.8±1.09	9.9±0.70	9.1±1.37	0.08
EF (%)	63.5±1.53	62.7±2.31	63.3±1.01	0.14

ED: erectile dysfunction; LVEDD: left ventricular end-diastolic diameter; LVESD: left ventricular end-systolic diameter; LA: left atrium; IVS: interventricular septum; PW: posterior wall; EF: ejection fraction.

The results of echocardiographic aortic elastic parameters are shown in Table 3 . The aortic strain values of Group 1, Group 2, and Group 3 were 8.38, 11.63, and 14.57, respectively, indicating statistically significant differences (p < 0.001). Aortic distensibility exhibited statistically significant variations among the different ED groups, with values of 0.60, 0.90, and 1.05 recorded for Group 1, Group 2, and Group 3, respectively. and 1.05 in Group 3 (p < 0.001). Similarly, there were statistically significant differences in both aortic systolic and diastolic diameter values between the ED groups (p < 0.001).

**Table 3:** Aortic elastic echocardiographic parameters of the ED groups

	Group 1 (severe ED) n = 24	Group 2 (moderate ED) n = 40	Group 3 (mild-moderate ED) n = 32	p value
AoSD (mm)	28±0.83	30.1 ±1.39	31.5±0.71	<0.001
AoDD (mm)	25.83±0.70	26.9±1.31	27.5±0.71	<0.001
Aortic strain (%)	8.38±1.45	11.93±2.45	14.57±2.01	<0.001
Aortic distensibility 10-3 cm^2^/dyn	0.60±0.18	0.90±0.38	1.05±0.28	<0.001

ED: erectile dysfunction; AoSD: aortic systolic diameter; AoDD: aortic diastolic diameter.

When the post-treatment changes in patient groups according to the severity of ED were examined, there were 24 patients in Group 1, 40 patients in Group 2, and 32 patients in Group 3 before treatment, while there were four patients in Group 1, 24 patients in Group 2, 16 patients in Group 3, 32 patients in Group 4, and 20 patients in Group 5 after treatment ( Table 4 ).

**Table 4:** Distribution of patients among erectile dysfunction groups before and after treatment

		Post-treatment groups					Total
		Group 1	Group 2	Group 3	Group 4	Group 5	
**Pre-treatment groups**	**Group 1**	4	16	4	0	0	24
	**Group 2**	0	8	12	20	0	40
	**Group 3**	0	0	0	12	20	32
**Total**		4	24	16	32	20	96

Group 1 (0-10, severe); Group 2 (11-16, moderate ED); Group 3 (17-21, mild-moderate ED); Group 4 (22-25, mild ED); and Group 5 (26-30, no ED).

The average delta IIEF score (difference between the pre-treatment and post-treatment IIEF scores) was determined as 5.13. While there was a high positive correlation between the patients' delta IIEF score and aortic strain (p < 0.01, r = 0.758), a moderate positive correlation was observed with aortic distensibility (p < 0.01, r = 0.574).

## Discussion

In this study, we aimed to investigate the role of AS in predicting the response to treatment in patients who were started on PDE-5 inhibitors for ED. Endothelial dysfunction and atherosclerosis are the most important etiological factors in CVD.^23^It is known that atherosclerosis affects cavernous vessels earlier than coronary arteries due to their smaller diameters. Endothelial dysfunction can also cause the development of ED; therefore, ED is considered an early manifestation of systemic vascular diseases.^15 , 24 , 25^Similarly, AS is used as a non-invasive method for the early detection of subclinical atherosclerosis. A decrease in the elasticity of the aorta and an increase in its stiffness are accepted as indicators of atherosclerotic change.^26^

In this study, we found that the values of aortic elasticity parameters, aortic strain, and aortic distensibility decreased in parallel with the severity of ED while AS increased. In a study by Demirelli et al. examining the relationship between the severity of ED and AS, there was an increase in the severity of ED as AS increased.^1^It is well known that NO plays an important role in the regulation of arterial elasticity. Thus, inhibition of the synthesis of NO increases AS. NO is one of the most important mediators in the physiology of erection.^10 - 12^The involvement of NO in the etiology of both ED and atherosclerosis suggests a relationship between these two conditions. Studies have also shown that patients with ED have a significantly higher AS than those without ED.^1 , 13 - 16^

Many epidemiological studies have demonstrated an association between ED and CVD. A meta-analysis revealed that men with ED had a significantly increased risk of CVD, coronary heart disease, and stroke compared to reference groups.^27 , 28^These associations can be attributed to endothelial dysfunction and the artery size hypothesis, which proposes that atherosclerosis affects all major vascular beds to the same extent and that penile arteries with a smaller diameter than the coronary and carotid vessels are affected earlier by atherosclerotic plaques of the same size. Systemic endothelial dysfunction may result in the penis not being able to regulate blood flow in the corpora cavernosis.^29^The most important finding of our study was the correlation between the patients' delta IIEF scores and aortic strain and distensibility, with a high level of positive correlation for the former and a moderate positive correlation for the latter. This suggests that AS is a parameter that can be used to predict the response to treatment prior to its initiation in patients with ED.

PDE-5 inhibitors have an important place in the treatment of ED. After the treatment of ED, the response is evaluated using the IIEF score. The high level of positive correlation between the delta IIEF score (i.e., the difference between the pre-treatment and post-treatment IIEF scores) and the aortic strain shows that AS can be evaluated with transthoracic echocardiography, which is easily applicable in predicting the response of patients with ED to PDE-5 inhibitors before the start of treatment. In addition, as mentioned above, since ED may be an early sign of systemic vascular diseases, a cardiac evaluation in these patients can assist the clinician in detecting possible CVD and predicting the response to PDE-5 enzyme inhibitors.

The limitations of our study are that it was single-center and the number of patients was small. There is a need for multicenter studies with larger case series on this subject.

## Conclusion

We determined that in patients with ED, aortic strain and aortic distensibility measured non-invasively using transthoracic echocardiography are important parameters in predicting patients' responses to PDE-5 inhibitor therapy.
